# Inside the Belly of the Beast: Exploring the Gut Bacterial Diversity of *Gonipterus* sp. n. 2

**DOI:** 10.1007/s00248-025-02524-1

**Published:** 2025-04-12

**Authors:** Rosa S. Knoppersen, Tanay Bose, Teresa A. Coutinho, Almuth Hammerbacher

**Affiliations:** 1 Department of Zoology and Entomology, Forestry and Agricultural Biotechnology Institute (FABI), University of Pretoria, Pretoria, 0002 South Africa; 2 Department of Biochemistry, Genetics and Microbiology, Forestry and Agricultural Biotechnology Institute (FABI), University of Pretoria, Pretoria, 0002 South Africa; 3 Centre for Microbial Ecology and Genomics, University of Pretoria, Pretoria, 0002 South Africa

**Keywords:** Eucalyptus snout beetle, Insect-microbe interactions, Invasive pests, Microbiome, Plant metabolites

## Abstract

The Eucalyptus snout beetle (*Gonipterus* sp. n. 2) is a destructive invasive pest of *Eucalyptus* plantations, responsible for significant defoliation and wood yield losses globally. Native to Australia, this beetle has adapted to thrive on diverse *Eucalyptus* hosts, overcoming their chemical defences. However, the mechanisms by which *Gonipterus* tolerates or utilises these plant defence metabolites remain poorly understood. In South Africa, *Gonipterus* sp. n. 2 poses a significant threat to *Eucalyptus* plantations by causing extensive defoliation and leading to substantial reductions in growth and wood production. This study investigates the relationship between diet, host *Eucalyptus* species, and the gut microbiome of *Gonipterus* sp. n. 2. Using controlled feeding experiments, beetles were reared on artificial, semi-artificial, and natural diets, as well as two *Eucalyptus* genotypes with distinct secondary metabolite profiles. High-throughput 16S rDNA sequencing and gas chromatography-mass spectrometry (GC–MS) revealed significant shifts in gut bacterial diversity and composition across diets. Natural diets supported the most diverse microbial communities, while artificial diets fostered a homogenised microbiome dominated by opportunistic taxa like *Serratia*. Host-specific effects were observed in frass microbiota, with substantial biotransformation of monoterpenes into less toxic derivatives. The results highlight the plasticity of *Gonipterus* gut microbiota, which enables metabolic adaptability and resilience in diverse environments. This microbial flexibility underpins the invasiveness of *Gonipterus*, emphasising the role of gut symbionts in overcoming host chemical defences. Understanding these interactions offers novel insights for microbiome-targeted pest management strategies, providing a sustainable approach to mitigate the impact of *Gonipterus* on global *Eucalyptus* forestry.

**Supplementary Information:**

The online version contains supplementary material available at 10.1007/s00248-025-02524-1.

## Introduction

The Eucalyptus snout beetle (*Gonipterus scutellatus *sensu lato; order—Coleoptera, family—Curculionidae) refers to a complex of 20 cryptic weevil species that affect a range of *Eucalyptus* hosts [1, 2]. Among the *Gonipterus* cryptic species complex, three are invasive, and include *G. pulverulentus*, *G. platensis* and the undescribed *Gonipterus* sp. n. 2 [1, 2]. These beetles are native to mainland Australia and Tasmania, but have been introduced to all major *Eucalyptus* growing regions globally, where these trees are an economically valuable source of wood, pulp, fibre and paper [2]. Adults are small, brownish, and have a distinctive rostrum. Females lay frass-covered egg packets with multiple eggs on leaves, where larvae hatch and feed, causing significant defoliation. After several instars, larvae pupate in the soil. The beetle’s life cycle spans 6–8 weeks, with multiple generations per year, making it a persistent *Eucalyptus* pest [2]. In South Africa, *Gonipterus* sp. n. 2 has caused substantial damage to *Eucalyptus* plantations through defoliation as well as damaging buds and stems [2]. It is estimated that prolonged feeding over several seasons by *Gonipterus* sp. n. 2 results in 20–50% of wood loss in *Eucalyptus* plantations [2, 3].

*Eucalyptus* produces a mixture of secondary metabolites rich in mono- and sesquiterpenes, phenolic compounds, and tannins [4]. Major volatile monoterpenes such as *α*-pinene, *d*-limonene, and 1,8-cineole are well-known plant secondary metabolites of *Eucalyptus* [5]. These volatiles can contribute to stress tolerance by mitigating thermal and oxidative stress [6]. Volatile monoterpenes may also play dual roles in insect-plant interactions. While most studies indicate that they function as deterrents, toxins and microbiocides, certain compounds, such as 1,8-cineole act as attractants, facilitating the beetle in locating its host [5, 7, 8]. In a previous study by Joubert et al. [9], certain *Eucalyptus* terpenes exhibited attractant activity, further aiding *Gonipterus* in finding new *Eucalyptus* hosts. Moreover, Branco et al. [10] and De Souza et al. [11] identified oxygenated terpenes from both the beetles’ emissions and their fras*s*, positing that these compounds could be possible semiochemicals. Evidence thus suggests that despite their toxicity, *Eucalyptus* monoterpenes and their metabolised products are utilised by *Gonipterus* for insect communication. However, the mechanisms by which the beetle detoxifies these plant secondary metabolites are not well understood in *Gonipterus*.

The gut microbiome of herbivorous insects facilitates the detoxification of plant secondary metabolites [12]. The gut microbial community can be classified as shared, found across insects within a population, or acquired, originating from the environment and diet of the insect [13]. Insect gut microbiomes facilitate detoxification by metabolising harmful plant secondary metabolites into less toxic and easily excretable products for the insect [12]. For example, in the red turpentine beetle, *Dendroctonus valens*, the gut microbiome assists in detoxifying the high concentrations of α-pinene in pine [14, 15]. Similarly, in the coffee berry borer, *Hypothenemus hampei*, gut bacteria such as *Pseudomonas*, demethylate caffeine into less toxic products [16]. However, it remains unknown how host species and chemical differences across plants affect the variation of insect gut microbiota. Understanding how microbial populations change with different diets may provide insights into how insects can overcome the secondary metabolites of their plant hosts.

Research on *Gonipterus* species is starting to uncover key members of their microbial community. For instance, various studies have identified *Beauveria bassiana*, an inhabitant and entomopathogen of the beetle, as an important fungus for controlling *Gonipterus* populations [17, 18]. Recently, Ribeiro et al. [19] conducted a study on the transmission of bacterial communities among *Gonipterus* eggs, adults and their parasitoids, finding bacterial associates playing a protective role in preventing parasitisation. *Serratia grimessi* was shared between *Gonipterus* eggs and adults. However, its function has yet to be explored [19]. Thus, the gut microbial community of *Gonipterus* remains poorly understood, and further research is needed to explore how diet, *Eucalyptus* species, and environmental factors influence this community. These studies will help clarify the role of symbionts in beetle health and behaviour, which is crucial for developing more effective pest management strategies.

This study aims to investigate the intricate relationship between diet including hosts with different leaf chemical composition, rearing environment, and the gut microbiome in *Gonipterus* sp. n. 2, focusing on both the composition of the bacterial community and its bioactivity. The first objective was to evaluate how varying rearing conditions, artificial, semi-artificial, and natural, affect the diversity and structure of the gut bacterial community. We predicted that beetles under natural rearing conditions would harbour a more diverse microbial community compared to those reared under artificial or semi-artificial conditions. The second objective was to examine the role of two different *Eucalyptus* host species, each with distinct secondary metabolite profiles, in shaping the gut and frass microbiomes of *Gonipterus* sp. n. 2. We hypothesised that the secondary metabolites present in *Eucalyptus* leaves would exert selective pressures on the microbial communities, leading to distinct microbial assemblages depending on the host plant species. The third objective was to assess the bioactivity of the gut microbiome by analysing volatile secondary metabolites in *Eucalyptus* leaves and frass, expecting microbial communities to metabolise plant-derived compounds, thereby altering the volatile profiles. Through these investigations, this study seeks to deepen our understanding of how rearing conditions, diet, and host plant interactions influence microbial dynamics in *Gonipterus* sp. n. 2, with potential implications for pest management strategies and microbial ecology.

## Materials and Methods

### Beetle Collection

**Fig. 1:**
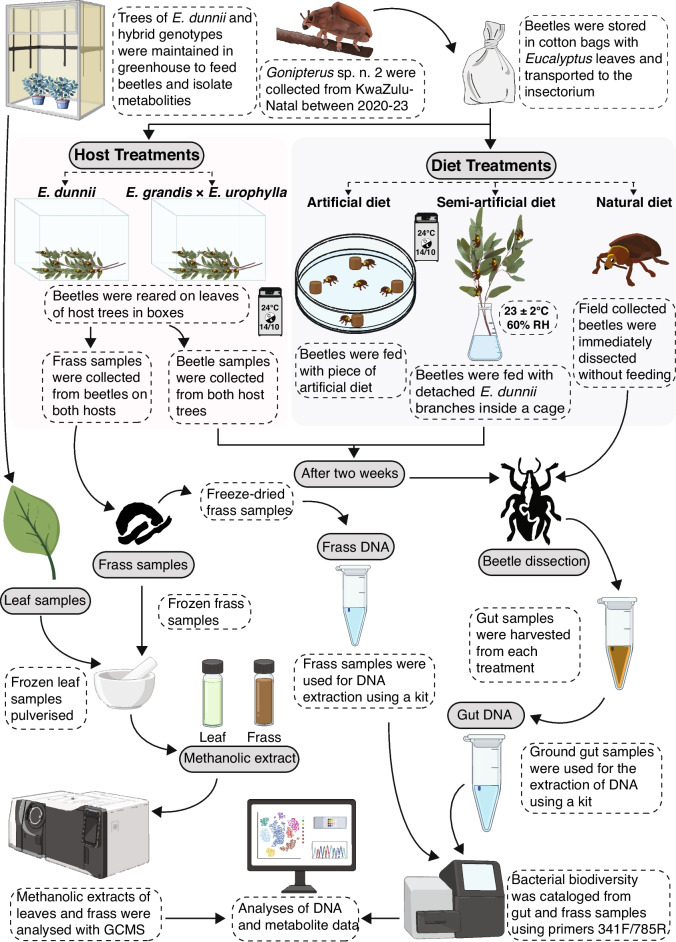
Experimental workflow to study the impact of various dietary treatments on bacterial biodiversity and plant metabolite breakdown within the gut of the *Eucalyptus* pest *Gonipterus* sp. n. 2

Between 2020 and 2023, adult *Gonipterus* sp. n. 2 from *Eucalyptus* spp. were collected from a private farm located in KwaZulu-Natal Province of South Africa (29.1934795° S 30.6082423° E). In the field, beetles were hand-picked from infested trees and were stored in cotton bags filled with *Eucalyptus* leaves inside a cooler box and transported to the insectarium of the Forestry and Agricultural Biotechnology Institute (FABI), University of Pretoria. At the facility, these field-collected beetles were divided into two categories, ‘diet’ and ‘host’ treatments (Fig. 1).

### Rearing of *Gonipterus* sp. n. 2 on Various Diets

#### Rearing Conditions

Three rearing conditions were implemented: artificial, semi-artificial, and natural diet (Fig. 1).

In the ‘artificial’ sub-group, beetles were reared for 2 weeks on a modified artificial diet formulated by Joubert et al. [9]. This diet consisted of two mixtures. Mixture 1 included 2.64 g of agar (Merck, Germany), 11.6 g of α-cellulose (Bio-Serv, USA), 8.60 g of casein (Bio-Serv, USA), 0.15 g of cholesterol (Bio-Serv, USA), 3.50 g corn starch (Robertsons, South Africa), 100 µl of linseed oil (Lemcke, South Africa), 0.78 g of Wesson salt mixture (MP Biomedicals, USA), 120 g of fresh *Eucalyptus dunnii* leaves in 200 ml of distilled water. Mixture 2 contained 0.25 g lecithin (MP Biomedicals, USA), 1.5 g of Vanderzant vitamins (Merck, Germany), 3 g of sucrose (Merck, Germany) and 0.16 g of ascorbic acid (Bio-Serv, USA) in a 40 ml of distilled water. Mixture 1 was autoclaved at 121 °C for 20 min. Mixture 2 was filter sterilised with a 0.22 µm Acrodisc® syringe filter (Pall Corporation, USA). After autoclaving, Mixture 1 was allowed to cool, after which Mixture 2 was added to reach a total volume of 240 ml. Preservatives included in the original recipe were omitted. The pieces of this sterilised solidified diet (5 mm in diameter) were distributed within Petri dishes containing 12 beetles. These setups were maintained inside a BINDER Series KBW 240 growth chamber (Baden-Württemberg, Germany) at a temperature of 24 °C and with a 14:10 h day-to-night cycle. In the ‘semi-artificial’ diet sub-group, beetles were reared for 2 weeks on cuttings of hedges of *E. dunnii* (cultivar DUN00) inside mesh cages measuring 600 × 40 cm. These cages were maintained at 23 ± 2 °C and relative humidity of 60 ± 2%. In the ‘natural’ rearing conditions sub-group, beetles were dissected immediately, without any prior feeding, to preserve the natural bacterial diversity present in the field (Fig. 1).

#### Host Treatments

The ‘host’ treatment was divided into two categories, *E. dunnii* (cultivar BE001) and *E. grandis* × *E. urophylla* (cultivar STGU1), based on the detached host tree leaves on which they were sustained for 2 weeks (Fig. 1). The leaves were changed thrice per week during the duration of the experiment. All the setups were maintained inside the BINDER Series KBW 240 growth chamber at a temperature of 24 °C and with a 14:10 h day-to-night cycle.

Leaves from both *Eucalyptus* cultivars were harvested from 4-year-old trees growing in 5 L plastic pots with pine bark potting mix (Culterra, South Africa). All trees were maintained under natural light conditions inside a greenhouse with a temperature of 23 ± 2 °C and relative humidity of 60 ± 2% at the experimental farm of the University of Pretoria (25.7472° S, 28.2588° E).

### Collection of *Eucalyptus* Leaf and Beetle Frass Samples

From the ‘host’ treatment setups, frass samples were also collected from beetles feeding on two different hosts. These samples were stored at − 80 °C for chemical analysis and DNA extraction (Fig. 1).

Eight fresh leaves from *E. dunnii* and *E. grandis* × *E. urophylla* were randomly collected from the apical part of the trees. The mid-ribs and the petioles were removed from all the leaves and the blades were individually flash-frozen using liquid nitrogen. All leaf tissue samples were stored at − 80 °C until metabolite extraction.

### Dissection of Beetle and Harvesting of Gut Samples

Sixty beetles were dissected after starving them for 24 h (Fig. 1). The elytra and wings were removed from all the beetles, after which beetles were surface sterilised with 0.05% (v/v) sodium hypochlorite solution for 15 s followed by 70% (v/v) ethanol for 15 s. All the beetles were rinsed twice with sterile deionised water. Beetles were fixed on wax-lined Petri dishes under sterile deionised water and dissected using a Nikon SMZ645 stereo microscope. Sterilised microdissection scissors were used to make an incision across the lining of the thorax and abdomen. The ends of the rectum and oesophagus were cut and carefully separated from the reproductive organs. Three guts were pooled for each of the treatment groups. All samples were immediately flash-frozen using liquid nitrogen and stored at − 80 °C until extraction of DNA.

### Extraction of DNA Beetle Gut and Frass Samples

#### Beetle Gut Samples

Twenty gut samples were macerated using a two-step approach. In the first step, samples were homogenised using a disposable pellet mixer attached to a cordless motor (VWR, Germany) in 1.5 ml microcentrifuge tubes. These tubes were incubated at 65 °C for 30 min. After this, samples were further pulverised using a Qiagen TissueLyser II at a frequency of 30 oscillations/sec for 5 min. The genomic DNA was extracted from pulverised samples using the QIAamp PowerFecal kit (Qiagen, Germany) following the manufacturer’s protocol (Fig. 1). The concentration and quality of DNA from the extracts were evaluated using a NanoDrop One microvolume UV–Vis spectrophotometer (Thermo Fisher Scientific, USA). The DNA samples were stored at − 80 °C.

#### Beetle Frass Samples

A total of 10 frass samples were collected, five each from both sub-groups of ‘host’ treatments (Fig. 1). Each 10 mg sample was lyophilised using a VirTis AdVantage Pro Freeze Dryer (SP Scientific, USA) for 24 h at a pressure of 13.33 mPa. Total DNA from frass samples was extracted with the Nucleospin Plant II kit (Macharey-Nagel, Germany). The frass samples were homogenised in 100 µl of PL 1 buffer from the kit using a disposable pellet mixer for 2 min, after which the remaining 300 µl of PL 1 buffer was added, and the mixture was incubated at 70 °C for 15 min. This mixture was further macerated using a Qiagen TissueLyser II for 5 min at a frequency of 30 oscillations/sec. Thereafter, the genomic DNA was extracted following the manufacturer’s protocol. DNA concentration and quality from extracts were assessed using the NanoDrop One microvolume UV–Vis spectrophotometer (Thermo Fisher Scientific, USA). All DNA samples were stored at − 80 °C.

### High-Throughput Sequencing of DNA

Bacterial biodiversity was catalogued from five gut DNA samples from each of three ‘diet’ treatments (5 samples/sub-group × 3 sub-groups = 15 samples), ten gut and five frass DNA samples from each sub-group of the ‘host’ treatment (10 gut samples × 2 sub-groups = 20 samples; 5 frass samples × 2 sub-groups = 10 samples; Fig. 1). The V3–V4 hypervariable region of the 16S rDNA was used as the marker gene region, which was amplified using the primers 341 F (5′–CCTACGGGNGGCWGCAG–3′) and 785R (5′–GACTACHVGGGTATCTAATCC–3′) [20]. Library preparation and paired-end Illumina MiSeq sequencing of DNA samples were outsourced to Macrogen, Inc., Seoul, South Korea. The raw Illumina sequence data files were deposited in the NCBI Sequence Read Archive (https://submit.ncbi.nlm.nih.gov/subs/sra/) under the accession number PRJNA1150032.

### Bioinformatic Analysis of DNA Sequence Data

The sequencing facility demultiplexed the raw data, which were then divided into three datasets: ‘diet’ treatment, gut from ‘host’ treatment, and frass from ‘host’ treatment. Each group was analysed separately. All three datasets were then analysed using two approaches: merging the forward and reverse reads and using only the forward reads. The paired-end reads were merged using BBMerge v38.97 [21]. However, during the merging and subsequent quality filtering with DADA2 [22], more than 50% of the reads were lost. Due to this, only the forward-read files were used for all three datasets.

Forward-read files were analysed using a bioinformatics pipeline available through Quantitative Insights into Microbial Ecology 2 (QIIME 2) v2023.6 [23]. The ‘q2-dada2’ plug-in was used for filtering, trimming, denoising, and removing singletons and chimeras. During this step, filtering parameters were set to a Phred quality score of 30 and a sequence length limit of 200 bp. Sequences that did not meet these criteria were excluded from further analysis. The ‘q2-vsearch’ package [24] was used for de novo clustering of reads into operational taxonomic units (OTUs) with 98% sequence similarity. Taxonomy assignment to OTUs was performed using the ‘qiime feature-classifier’ [25] using the SILVA 138 SSURef NR99 bacterial database [26], trained with primers 341 F and 785R. OTUs of mitochondrial and chloroplast origins were manually deleted from the final taxonomic table.

### Statistical Analysis of DNA Sequence Data

The taxonomic hierarchical plots were constructed using Flourish (https://flourish.studio/). Order was used as the preferred taxonomic level for statistical analyses of all three datasets using Microbiomeanalyst 2.0 [27]. Low-count features were excluded based on their mean abundance, applying a minimum threshold of 4. Features with low variance were removed by considering the interquartile range. The remaining data were normalised using total sum scaling (TSS). α diversity was assessed using the Shannon and Simpson indices, while species richness was estimated with the Chao1 metric. For the β diversity, PCoA plots were generated using the Bray–Curtis index for β diversity and statistical significance was evaluated with PERMANOVA. If the PCoA failed to show distinct clustering of data points, but PERMANOVA revealed statistically significant results, PERMDISP was used to confirm whether the observed differences were driven by data dispersion rather than variations in community structure.

### Extraction of Metabolites from Leaf and Frass Samples

Frozen leaf samples were individually pulverised using a sterile mortar and pestle with liquid nitrogen (Fig. 1). Four samples of leaf tissue (45 mg) from each genotype were added to 1.5 ml methanol (Sigma-Aldrich, USA) and agitated continuously for 1 h on an orbital shaker. Homogenates were centrifuged at 10,000 rpm for 10 min, and the supernatant was transferred into screw cap glass vials. Methanolic extracts of leaves were analysed using an Agilent 7890 gas chromatograph equipped with a quadrupole mass spectrometer (GCMS; Agilent Technologies, USA) using a 30 m × 0.25 mm × 0.25 µm ZB-WAX column (Phenomenex, USA). One µl of the sample was injected directly using a split mode of 10:1. The GC oven gradient was set to 10 °C min^−1^, from an initial temperature of 50 °C to a maximum temperature of 250 °C. The flow rate of the carrier gas (helium) was set for 1.2 ml min^−1^. MS settings were adjusted to scan a mass range between 40–350 *mz*^−1^. Metabolite extraction of frass was done using the abovementioned protocol, except that 20 mg frozen frass was added to 0.80 ml methanol (Agilent Technologies, USA) (Fig. 1).

### Analysis of Metabolite Data from Leaf and Frass Samples

The raw GC–MS data from the *Eucalyptus* genotypes was converted from. d to. mzXML format using Proteowizard [28]. This data was then processed using XCMS Online (https://xcmsonline.scripps.edu/), where multigroup statistical analysis was conducted based on the *Eucalyptus* genotype [29]. From the XCMS results table, base peak height, mass, and retention times were extracted. Data features with a mean value below 1000 were removed from the dataset. To compare the chemical profiles of plant and frass groups as well as the different *Eucalyptus* genotypes from ‘host’ treatment beetles feeding on two *Eucalyptus* hosts, the extracted data underwent normalisation by sum, quantile normalisation and log-transformation for multivariate analysis using MetaboAnalyst 6.0 (Pang et al., 2021).

Additionally, *Eucalyptus* genotypes and the associated beetle frass GCMS data were further processed in MassHunter Unknowns Analysis software vB.09.00 (Agilent Technologies, USA). Peak data was deconvoluted with the following parameters, left *m/z* delta at 0.3, right *m/z* delta at 0.7 and a sharpness threshold of 25%. A peak filter was not added, and the compound identification minimum match factor was set at 30. Deconvoluted data was exported, manually curated, and uploaded into MetaboAnalyst 6.0 [30]. Data of *Eucalyptus* leaves and frass were normalised through autoscaling. A heatmap was generated to show differences in major monoterpene concentrations between *Eucalyptus* genotypes and frass from beetles feeding on these genotypes.

## Results

### Bacterial Biodiversity Associated with Guts of *Gonipterus* sp. n. 2 Reared Under Different Conditions

**Fig. 2:**
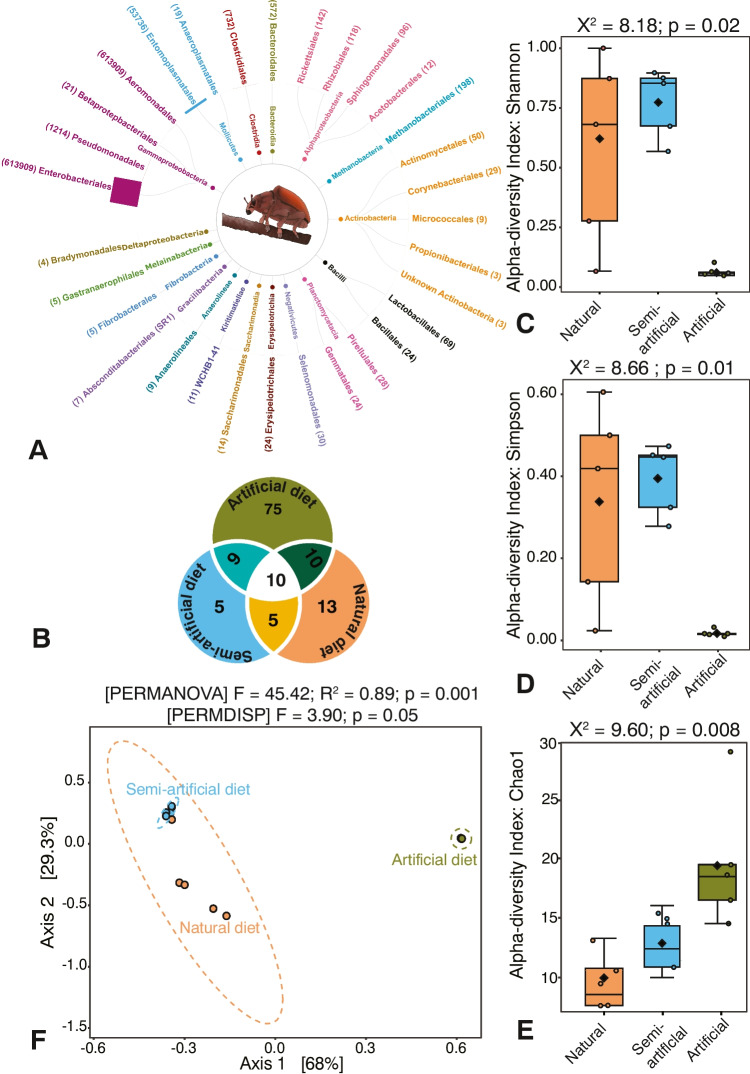
Effects of diet and rearing environment on gut-associated bacterial biodiversity in *Gonipterus* sp. n. 2. **A** Taxonomic composition of bacteria in the gut up to order level. Numerical within the parenthesis beside taxon names indicate read number; **B** Venn diagram showing the number of shared and unique OTUs between the three diet and rearing environment sub-groups; **D** Shannon index, **E** Simpson index, **F** OTU richness (Chao1), and (**G**) principal coordinates analysis. Analyses of α and β diversity were conducted in Microbiomeanalyst 2.0. α diversity (Shannon, Simpson) and species richness (Chao1) were assessed on original data using Kruskal–Wallis tests. β diversity was analysed with normalised data using a Bray–Curtis based PCoA and evaluated with PERMANOVA and PERMDISP

A total of 797,295 raw reads were obtained from 15 gut DNA samples of *Gonipterus* sp. n. 2, representing three rearing conditions: ‘artificial’, ‘semi-artificial’, and ‘natural’. Following quality filtering and chimera removal, 673,519 high-quality reads (84.24%) were retained for downstream analysis. After the taxonomic assignment, OTUs originating from chloroplast, mitochondria and low-count taxa were removed, leaving 670,999 (84.16%) reads corresponding to 126 OTUs (Supplementary Table 1). These OTUs represented 30 well-defined bacterial orders, along with two phylotypes, WCHB1 - 41, and B12-WMSP1, and two undescribed lineages of Actinobacteria and Firmicutes (Fig. 2A). The five most abundant bacterial orders were Enterobacteriales (91.49%), Entomoplasmatales (8.01%), Pseudomonadales (0.18%), Clostridiales (0.11%), and Bacteroidales (0.09%) (Supplementary Table 1), with the dominant genera being *Serratia* (36.41%), *Rahnella* (29.59%), an unidentified Enterobacteriaceae (24.56%), *Mesoplasma* (8.01%), and *Erwinia* (0.94%). Shared OTUs among three diet sub-groups were limited. Ten OTUs were shared across all three treatment groups (Fig. 2B). The ‘artificial’ diet group exhibited the highest number of exclusive OTUs, highlighting the distinct microbial composition associated with this diet (Fig. 2B).

The α diversity indices revealed significant differences in biodiversity across ‘natural’, ‘semi-artificial’, and ‘artificial’ sub-groups. The Shannon index (*p* = 0.02) was highest in the ‘natural’ sub-group, reflecting substantial diversity and variability, followed by the ‘semi-artificial’ rearing condition sub-group with moderate diversity and reduced variability. The artificial diet sub-group exhibited the lowest Shannon index values, indicating minimal diversity and a homogenous community structure (Fig. 2C). Similarly, the Simpson index (*p* = 0.01) showed a clear gradient, with the ‘natural’ rearing condition sub-group having the highest diversity, the ‘semi-artificial’ diet sub-group showing moderate diversity, and the ‘artificial’ diet sub-group displaying near-zero values (Fig. 2D). The richness index, Chao1 (*p* = 0.008), revealed a different pattern, with the ‘artificial’ diet sub-group exhibiting the highest richness compared to ‘semi-artificial’ and ‘natural’ rearing conditions (Fig. 2E).

The PCoA plot illustrates clear separation among bacterial communities associated with ‘natural’, ‘semi-artificial’, and ‘artificial’ rearing conditions sub-groups. Axis 1, which explains 68% of the total variation, primarily drives the differentiation, while Axis 2 accounts for an additional 29.3% (Fig. 2F). Bacterial communities associated with natural and semi-artificial rearing conditions clustered closely together but remained distinct. In contrast, communities associated with artificial diets formed a separate cluster, indicating substantial compositional differences (Fig. 2F). PERMANOVA confirmed significant differences among the groups (*p* = 0.001), with the majority of the variance explained by the rearing environment type. PERMDISP analysis suggests no significant differences in dispersion across groups (*p* = 0.05), indicating that the observed separation is not due to heterogeneity within groups (Fig. 2F).

### Metabolite Composition of *Eucalyptus* Hosts and Frass

The bacterial biodiversity data showed that diet plays a significant role in shaping the gut bacterial communities of *Gonipteru*s sp. n. 2. To assess how gut bacterial communities were influenced by chemical composition, we analysed metabolites in two *Eucalyptus* hosts and the frass of *Gonipterus* sp. n. 2 feeding on them.

**Fig. 3:**
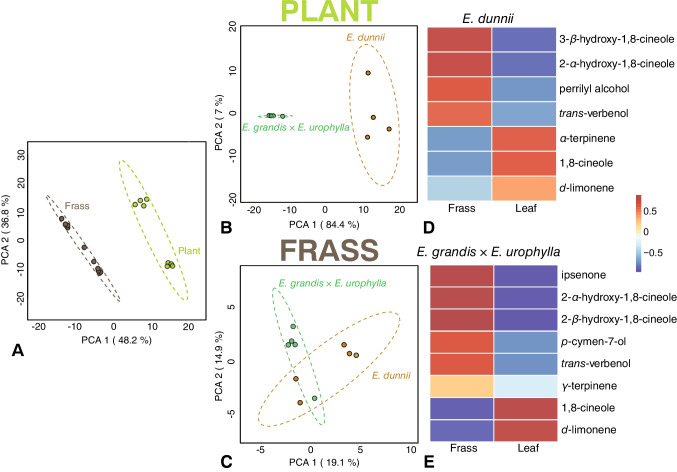
Foliar volatile content of *Eucalyptus* genotypes and *Gonipterus* frass. (**A**) Relative GCMS intensities of metabolites between plant and frass groups (**B**) Relative GC–MS peak intensities of volatile organic compounds in leaves of two *Eucalyptus* genotypes: *Eucalyptus dunnii* and *Eucalyptus grandis* × *Eucalyptus urophylla*. (**C**) Relative GC–MS peak intensities of volatile organic compounds in beetle frass after beetles consumed *E. dunnii* or *E. grandis* × *E. urophylla* host leaves. (**D**, **E**) Heatmap showing the relative concentrations of specific monoterpenes in *Eucalyptus* leaves and their biotransformation products in beetle frass after beetles were fed on leaves from *E. dunnii* and *E. grandis* × *E. urophylla* hosts. Data were normalised and log-transformed in Metaboanalyst 6.0 for PCoA generation. Additional processing and deconvolution of *Eucalyptus* genotypes and the associated frass samples were conducted in Mass Hunter, followed by normalisation and heatmap generation to compare the relative concentrations of metabolites across the samples

To determine the difference between *Eucalyptus* and frass metabolite profiles as well as the volatile metabolite profiles of *E. dunnii* and *E. grandis* × *E. urophylla* were analysed using GC–MS. The PCAs between *Eucalyptus* and frass and *Eucalyptus* hosts revealed distinct chemical differences (Fig. 3A and B). Beetles were reared on leaves from each genotype, and their frass was collected for metabolite analysis. A PCA of frass metabolites showed considerable overlap between the two genotypes (Fig. 3C), indicating that host-specific chemical differences may diminish during digestion.

Comparisons of monoterpene profiles between leaves and frass revealed significant reductions in major compounds, such as 1–8 cineole and* d*-limonene, in the frass from both hosts (Fig. 3D and E). In contrast, hydroxylated and oxygenated terpene derivatives were elevated in the frass. Three oxygenated derivatives of 1,8-cineole, 2-α-hydroxy- 1,8-cineole, and 3-β-hydroxycineole, were abundant in the frass (Fig. 3D and E). Biotransformed products such as perillyl alcohol (from* d*-limonene) and *trans*-verbenol (an oxygenated form of α-pinene) were detected in high concentrations in the frass (Fig. 3D and E), suggesting active biotransformation of plant secondary metabolites during beetle digestion.

### Bacterial Biodiversity Associated with Gut and Frass of *Gonipterus* sp. n. 2 from ‘Host’ Treatments

Twenty gut and ten frass samples were barcoded to catalogue the bacterial biodiversity associated with *Gonipterus* sp. n. 2 feeding on the leaves of two host trees, *E. dunnii* and *E. grandis* × *E. urophylla*.

#### Gut Samples

**Fig. 4:**
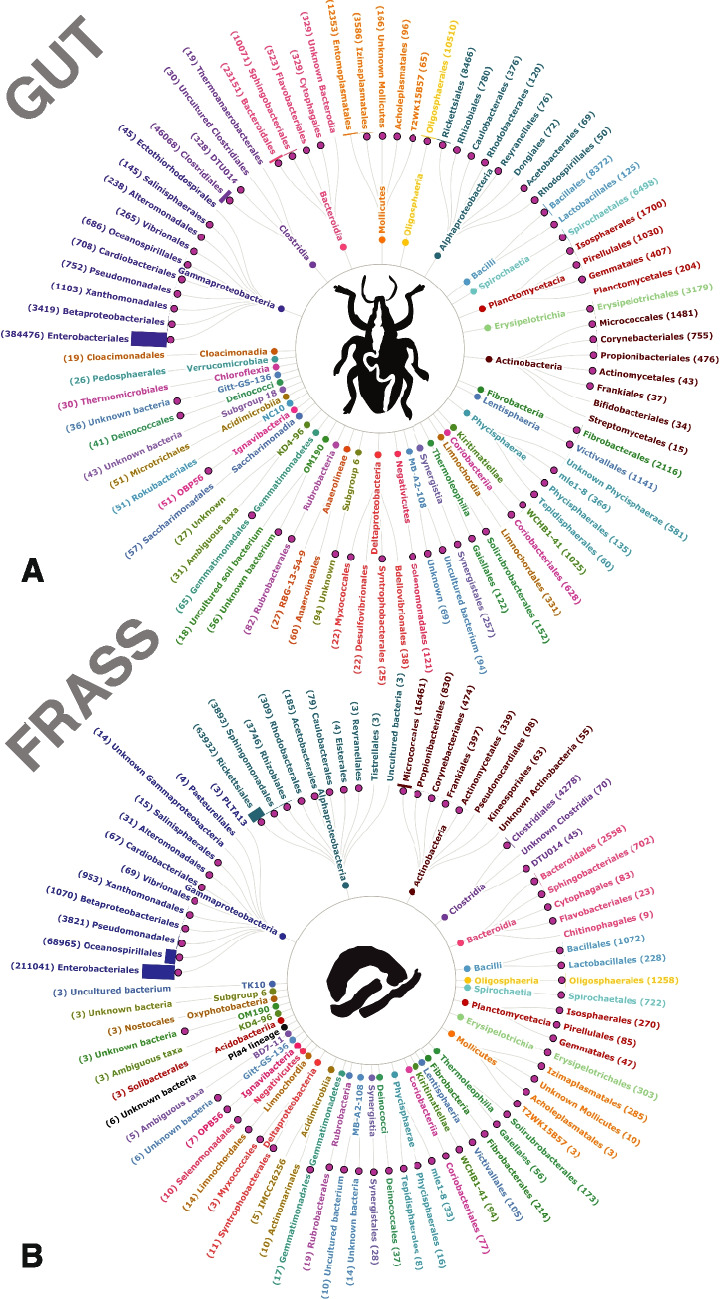
Radial plot illustrating the taxonomic composition of bacteria in the gut and frass of *Gonipterus* sp. n. 2 after feeding on *E. dunnii* and *E. grandis* × *E. urophylla*. The plot was generated in Flourish. The numerical within the parenthesis beside each taxon indicates the read number. Overlapping taxa are marked with purple bullets

A total of 791,200 raw reads were obtained from the gut DNA of 20 samples. After quality filtering, 609,879 high-quality reads (77.08%) were retained for downstream analyses. Following taxonomic assignments, OTUs associated with chloroplast, mitochondrial and reads with low counts were deleted, which resulted in a final dataset that had 532,720 reads (67.33%, Supplementary Table 2). These reads corresponded to 345 bacterial OTUs spanning across 73 well-defined orders, ten phylotypes, such as OPB56, Gitt-GS- 136, RBG- 13–54 - 9 and others, and six unknown OTUs representing various taxonomic groups (Fig. 4A; Supplementary Table 2). The dominant bacterial orders identified in the gut samples were Enterobacteriales (69.72%), Clostridiales (8.28%), Bacteroidales (4.19%), Entomoplasmatales (2.24%), and Oligosphaerales (1.91%) (Fig. 4A; Supplementary Table 2). At the genus level, the most abundant taxa included an undescribed genus within Enterobacteriaceae (43.79%), *Hafnia*-*Obesumbacterium* (25.69%), *Erwinia* (6.38%), *Hydrogenispora* (3.22%), and *Mesoplasma* (2.24%).

**Fig. 5:**
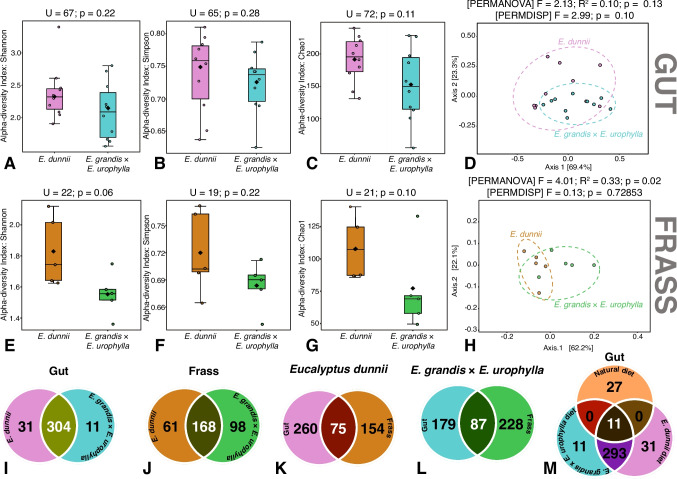
Effects of feeding on *E. dunnii* and *E. grandis* × *E. urophylla* leaves on gut and frass-associated bacterial biodiversity in *Gonipterus* sp. n. 2. gut (**A**–**C**) α diversity indices; **D** principal coordinates analysis. Frass (**E**–**G**) α diversity indices; **H** principal coordinates analysis. Venn diagrams illustrating shared and unique OTUs between (**I**) the gut of beetles fed with E*ucalyptus dunnii* or *E. grandis* × *E. urophylla*, (**J**) the frass of beetles fed with *E. dunnii* or *E. grandis* × *E. urophylla*, **K** the gut and frass of beetles fed with *E. dunnii*. **L** The gut and frass of beetles fed with *E. grandis* × *E. urophylla*. **M** The gut of beetles fed with *E. dunnii*, *E. grandis* × *E. urophylla*, or the ‘natural’ sub-group from the field (see Fig. 1). α and β diversity analyses were performed using MicrobiomeAnalyst 2.0. α diversity was assessed using original data and Mann–Whitney tests. β diversity was analysed using a Bray–Curtis-based PCoA on filtered and normalised data, with statistical significance evaluated with PERMANOVA and PERMDISP

The diversity analysis of gut microbiota of *Gonipterus* sp. n. 2 feeding on leaves of *E. dunnii* and *E. grandis* × *E. urophylla* revealed no statistically significant differences in either α or β diversity metrics. α diversity indices, such as Shannon (*p* = 0.22; Fig. 5A), Simpson (*p* = 0.28; Fig. 5B), and Chao1 (*p* = 0.11; Fig. 5C), showed comparable microbial richness and evenness across groups. In the PCoA plot, the data points emerging from beetle guts feeding on two different host trees overlapped, suggesting high within-group variability (Fig. 5D). Likewise, PERMANOVA (*p* = 0.13) indicates no significant compositional differences, while PERMDISP (*p* = 0.10) suggests marginal but non-significant differences in dispersion (Fig. 5D).

#### Frass Samples

From 10 DNA samples extracted from the frass of *Gonipterus* sp. n. 2 feeding on *E. dunnii* and *E. grandis* × *E. urophylla*, a total of 692,467 reads were recovered. After quality filtering, 572,550 reads (82.68%) were retained. Reads identified as originating from plant mitochondria chloroplasts (33.53%) low read counts were excluded following taxonomic assignment, leaving 390,176 (56.34%) bacterial reads that clustered into 331 OTUs representing 63 well-defined bacterial orders, several phylotypes and previously unknown bacteria (Fig. 4B). The predominant bacterial orders in the beetle frass samples were Enterobacteriales (64.68%), Oceanospirillales (21.14%), Micrococcales (5.03%), Clostridiales (1.91%), and Sphingomonadales (1.19%) (Fig. 4B; Supplementary Table 3). At the genus level, the most abundant taxa were an unidentified genus within Enterobacteriaceae (51.31%), *Hafnia* (19.04%), *Carnimonas* (21.14%), *Pseudomonas* (1.16%), and *Methylobacterium* (0.77%).

The analysis of frass bacterial microbiota revealed significant differences in α and β diversity between beetles feeding on *E. dunnii* and *E. grandis* × *E. urophylla* leaves. α diversity, as measured by the Shannon index, showed significantly higher bacterial diversity in the frass of beetles feeding on *E. dunnii* (*p* = 0.06; Fig. 5E) but not the Simpson index (*p* = 0.22; Fig. 5F). Chao1 index indicated a slightly greater bacterial richness in the frass of beetles feeding on *E. dunnii* leaves (*p* = 0.10; Fig. 5G). In the PCoA plot, data points from both treatments overlapped (Fig. 5H). However, PERMANOVA confirmed significant differences in microbial community composition between the two treatments (*p* = 0.02). PERMDISP analysis (*p* = 0.73) indicated that these differences were due to compositional variation rather than dispersion within groups (Fig. 5H).

### Pairwise Comparison of Bacterial Biodiversity Between Gut and Frass Samples

To illustrate the shared and unique OTUs in the gut and frass microbiota of *Gonipterus* sp. n. 2 feeding on *E. dunnii* and *E. grandis* × *E. urophylla*, Venn diagrams were constructed. In the gut microbiota, 304 OTUs were shared between beetles feeding on the two hosts, with 31 and 11 unique OTUs identified in *E. dunnii* and *E. grandis* × *E. urophylla*, respectively (Fig. 5I). In the frass microbiota, 168 OTUs were shared, while beetles feeding on *E. dunnii* had 61 unique OTUs, compared to 98 unique OTUs for those feeding on *E. grandis* × *E. urophylla* (Fig. 5J). Within *E. dunnii*, 75 OTUs were shared between the gut and frass, with 260 and 154 OTUs unique to the gut and frass, respectively (Fig. 5K). Similarly, within *E. grandis* × *E. urophylla*, 87 OTUs overlapped between the gut and frass, while 179 and 228 OTUs were exclusive to the gut and frass, respectively (Fig. 5L).

Gut bacterial OTUs were compared between beetles from the ‘natural’ diet sub-group that were dissected immediately after collection to represent wild feeding and beetles from the ‘host’ treatment, fed for 2 weeks on detached branches of *E. dunnii* or *E. grandis* × *E. urophylla* (Fig. 5M). Only 11 bacterial OTUs were shared across the three treatments. No OTUs were shared between beetles on the ‘natural’ diet and those feeding on *E. dunnii* or *E. grandis* × *E. urophylla* in the lab. However, 293 OTUs were shared between ‘lab-reared’ beetles fed on *E. dunnii* and *E. grandis* × *E. urophylla* (Fig. 5M).

## Discussion

This study explored the combined effects of rearing conditions, host *Eucalyptus* species, and bioactivity of the gut and frass bacterial communities of *Gonipterus* sp. n. 2, using controlled rearing experiments, high-throughput sequencing, and metabolite profiling. Beetles were exposed to three rearing conditions, ‘natural’, ‘semi-artificial’, and ‘artificial’, and two host treatments involving rearing the beetles on *E. dunnii* and an *E. grandis* × *E. urophylla* hybrid genotype. Significant shifts in bacterial diversity and composition were observed, with natural diets supporting the highest diversity and artificial diets fostering a more homogenised bacterial community. Host-specific effects were most pronounced in the frass bacterial community, with bacterial richness and composition differing significantly between hosts. Complementing these microbial analyses, metabolite profiling of leaves and frass revealed substantial biotransformation of host plant secondary metabolites during digestion. Terpenes such as 1,8-cineole and *d*-limonene were significantly reduced in frass, while hydroxylated and oxygenated derivatives, including 2-α-hydroxy- 1,8-cineole and *trans*-verbenol, were enriched, indicating active microbial and beetle metabolism.

### Rearing Conditions and Diet Significantly Influence the Gut Bacterial Communities in *Gonipterus* sp. n. 2

We compared the effects of different rearing conditions and diets on the gut bacterial community of *Gonipterus* sp. n. 2. The results showed a significant differentiation in gut bacterial communities between beetles reared under ‘artificial’, ‘semi-artificial’ and ‘natural’ rearing conditions. Beetles feeding on an artificial diet in captivity exhibited gut bacterial communities dominated by a single taxon, *Serratia*. This dominance of a specific bacterial genus, alongside the reduced bacterial diversity, aligns with patterns of insect-microbial dysbiosis. In contrast, beetles feeding on *Eucalyptus* hosts from the ‘natural’ and ‘semi-artificial’ conditions showed overlapped but distinct gut bacterial communities. Such differentiation suggests that diet, as well as the rearing environment, might be involved in structuring the gut bacterial communities of *Gonipterus* sp. n. 2.

In beetles from the ‘artificial’ diet group, OTUs from the genus *Serratia* were highly abundant. Although *Serratia* is commonly found in the gut microbiome of various insects, it is rarely over-abundant [31]. Several studies suggest that *Serratia* can facilitate important functions within the insect, such as the detoxification of plant secondary metabolites [32]. However, when abundant, it can become an opportunistic pathogen of the insect host [31]. Mason et al. [33] studied this phenomenon in noctuid Lepidoptera, *Spodoptera* and *Helicoverpa*, revealing that variations in dietary nutritional concentrations can alter the relationship of *Serratia*, shifting it from mutualistic to pathogenic.

The disruption of the gut bacterial community in beetles on the ‘artificial’ diet can be attributed to its high carbohydrate content and low concentrations of *Eucalyptus* volatiles (Supplemental material 1). *Eucalyptus* monoterpenes, like that of 1,8-cineole, *d*-limonene and *α*-pinene, may play significant roles in the shaping of the gut bacterial community. These volatiles have anti-microbial properties that could suppress the growth of unwanted bacterial communities while supporting communities with plant secondary metabolite detoxification abilities [34]. As a result, high levels of purified carbohydrates, coupled with the absence of *Eucalyptus* volatiles, may enable the proliferation of restricted microbial populations that are typically suppressed by these volatiles. Similar findings have been reported in American cockroaches (*Periplaneta americana*), where synthetic diets rich in purified carbohydrates caused microbial community imbalances, leading to reduced bacterial diversity and abundance of rare species [35].

While ‘artificial’ rearing conditions are valuable tools for studying insect and insect-microbial interactions, they are not always viable alternatives to the natural habitat of an insect [36]. Artificial diets often fail to replicate the physical and chemical complexity of natural diets, which can lead to significant changes in the gut bacterial community [36]. In this case, the low abundance of *Eucalyptus* volatiles and the increase in carbohydrates in the artificial diet resulted in the overgrowth of bacterial communities that included opportunistic pathogens. *Gonipterus* sp. n. 2 has a preference for *Eucalyptus* leaves with high levels of 1,8-cineole [9], and these volatiles could thus facilitate the maintenance of a stable gut bacterial community and indirectly support the immune function of the beetles.

### The Host Plant’s Chemical Composition Does Not Significantly Influence the Gut Bacterial Community Composition in *Gonipterus* sp. n. 2

Following the outcome of the ‘diet’ treatment, we investigated if the gut bacterial community of *Gonipteru*s sp. n. 2 would vary between groups of beetles feeding on two common *Eucalyptus* hosts in South Africa. Our metabolite analysis showed these two *Eucalyptus* varieties have a distinct chemical profile. We recorded a high bacterial diversity from the guts of beetles feeding on both the hosts yet there were no significant differences. These findings contrast with previous results, which showed that diet plays a major role in shaping the gut bacterial community and the rearing environment. Bacterial communities associated with the frass from beetles feeding on the different *Eucalyptus* hosts revealed a slight shift in chemical content and bacterial community composition. However, while this may be the case, the amount of dispersion between the bacterial community of the frass does not allow for a clear interpretation.

Similar patterns of gut bacterial community stability have been observed in *Lymantria dispar* and Cerambycid beetles, where dietary changes had minimal impact on the composition of the gut microbiota [37, 38]. However, it contrasts with many more studies that demonstrate that diet has a significant influence on the gut bacterial community [39–41]. For instance, findings from tropical leaf beetles show that the shift to exotic plants significantly changed gut bacterial communities, thus facilitating niche adaptation [42]. These discrepancies highlight the complexity of bacterial responses to dietary changes and suggest that factors such as plant hosts, exposure duration, and gut bacterial load play pivotal roles.

Recent research highlights the pivotal role of plant-associated microbiota and secondary metabolites in shaping insect gut microbiomes [43]. Although it is well known that insects rely on microbiomes, identifying the drivers for microbiome variation remains challenging [43] because insects can interact with plant-associated microbes through two primary pathways. These are directly via feeding on plant tissues or indirectly through the plant’s chemical profile affecting microbial activity [44]. These interactions remain relevant for *Gonipterus*, an oligophagous herbivore, as the microbial communities associated with its host plants may significantly influence the composition and function of its gut microbiota [42].

In the case of *Gonipterus* sp. n. 2, the observed increase in bacterial diversity and minimal variation in the bacterial community suggest that its gut microbiota is highly adaptable to changes in diet. However. the stability of shared bacterial OTUs within the gut microbiota suggests the presence of selective pressures that enable the beetle to preserve functionally essential microbial communities. Over time, these pressures may help stabilise the gut bacterial community, allowing it to revert to a more balanced state.

Field observations may give further context to the lack of variation and increase in bacterial diversity in the gut of *Gonipterus* sp. n. 2. Beetles exhibit a preference for remaining on their current *Eucalyptus* host rather than migrating to new ones. However, when the population on a single tree becomes too large, they relocate to adjacent *Eucalyptus* hosts (*pers. obs.*). Various studies have indicated that when insects increase their range of diets, populations of symbionts can proliferate and have adverse effects on insect immunity [45, 46]. Consequently, it has been found that insects transcribe elevated basal levels of immunity-related genes that provide a protective effect against bacterial infections during diet shifts [45]. Within this period, the beetle may undergo an unstable transitional period wherein the immune system is compromised and can result in the colonisation of opportunistic pathogens [45].

Successful host plant colonisation can also be attributed to the acquisition of new microbial associates [37]. Herbivorous insects are exposed to a difficult-to-digest, nutrient-poor diet coupled with a chemical defence system that renders the diet less nutritious [47]. Consequently, insects have different strategies for overcoming these challenges [47]. One such way is by acquiring microbial communities that facilitate the digestion of the plant host tissues [47]. Many of these interactions can be observed across insect species [48]. For example, *Cassida rubiginosa*, the thistle tortoise beetle, hosts *Stammera*, a bacterium that produces crucial pectinases that are required for the digestion of its diet [49]. Similarly, *Hypothenemus hampei*, the coffee berry borer, contains several gut bacteria, such as *Pseudomonas parafulva*, that facilitate the detoxification of caffeine that is abundant in coffee beans [16, 50]. A comparable example is found in the moth *Hyphantria cunea*, where the gut bacterial community is believed to help in the detoxification of plant secondary metabolites, facilitating host expansion [51].

### Potential Roles of Gut Bacterial Community in *Gonipterus* sp. n. 2

The most abundant bacterial families shared between the ‘host’ treatment and the ‘semi-artificial’ sub-group from the ‘diet’ treatment were *Enterobacteriaceae* and *Entomoplasmataceae*. The prominent genera identified within these families included *Hafnia/Obesumbacterium*, *Rahnella*, and *Entomoplasma*. *Hafnia/Obesumbacterium* and *Rahnella* are related taxa that appear to replace each other across different beetle treatments. While the specific function of *Hafnia/Obesumbacterium* remains unknown, *Rahnella* is a common symbiont known for its ability to fix nitrogen and detoxify plant secondary metabolites [52]. *Entomoplasma*, on the other hand, is not known to be as widespread among insects, and as such is not well studied [53].

While little is known about *Entomoplasma*, they are generally categorised as pathogenic bacterial reproductive manipulators, similar in function to *Wolbachia* and *Spiroplasma* [54]. However, new research on leaf-cutting ants from genera *Atta* and *Acromyrmex* revealed that members of *Mesoplasma*, which are closely related to *Entomoplasma* are vertical symbionts that can play pivotal roles as nutritional mutualists [55, 56].

### Effect of the Gut Microbiome on the Invasiveness of *Gonipterus*

Several species of *Gonipterus* are important invasive pests, threatening *Eucalyptus* plantations in diverse climatic regions worldwide [2, 57]. Their success as global pests stems from their ability to thrive across different environmental conditions and adapt to various host *Eucalyptus* species. In this study, we used *Gonipterus* sp. n. 2 as a model organism and revealed that the gut bacterial community of the beetle exhibits noticeable flexibility. This adaptability enables *Gonipterus* to process a broad range of diets, an essential trait for survival in non-native environments where their preferred host trees may not always be available.

This microbial plasticity is a hallmark of many successful invasive species, allowing them to overcome dietary or environmental constraints in new territories. For instance, the polyphagous nature of the fall armyworm, *Spodoptera frugiperda*, is partly attributed to gut microbiota that enhances its ability to digest diverse plant compounds [58]. Similarly, the red imported fire ant, *Solenopsis invicta*, relies on microbial symbionts to exploit a variety of resources in its invasive range [59, 60], so as the small hive beetle,* Aethina tumida* [61]. A similar trend has also been reported from various invasive plants where they reshape the soil microbial community and structure and soil properties to displace native flora and establish themselves in a wide range of climatic regions worldwide [62, 63]. Likewise, the flexible gut microbiome of *Gonipterus* likely facilitates its dietary expansion, aiding its establishment and proliferation in varied ecological niches.

The above examples highlight the integral role of host-microbiome interactions in invasion biology, emphasising their contribution to the ecological success of invasive species across taxa. Thus, understanding the role of the gut-associated microbiome in *Gonipterus* invasions can also provide critical insights for managing its impacts on the global *Eucalyptus* forestry landscape. Microbiome-targeted strategies, such as those explored for controlling *Spodoptera* [64] and various other pests [65–68], could offer novel approaches for managing *Gonipterus* populations in the future. Potential applications include the selective breeding of *Eucalyptus* trees with enhanced resistance to suppress the growth of the beneficial microbiome associated with *Gonipterus*. Additionally, microbial detoxification products could be leveraged in attract-and-kill strategies to sustainably reduce the impact of *Gonipterus* on the *Eucalyptus* forestry industry.

## Conclusions

Our study highlights the complex interplay between diet, rearing conditions, and the gut microbiome in shaping the ecological success of *Gonipterus* sp. n. 2. The adaptability of its gut bacterial community underpins the ability of the beetle to thrive on diverse *Eucalyptus* hosts across varied environments, a key factor contributing to its status as a successful invasive pest. By maintaining shared bacterial taxa and adjusting community composition in response to dietary shifts, *Gonipterus* ensures both metabolic flexibility and ecological resilience. These findings align with broader patterns observed in invasive species, where host-microbiome interactions play a pivotal role in overcoming environmental challenges and facilitating niche expansion.

The insights gained from this study extend beyond *Gonipterus*, offering a comparative framework for understanding microbial plasticity in other invasive species, such as *S. frugiperda* and *S. invicta*. Similar mechanisms, including microbial facilitation of detoxification and nutrient acquisition, are observed across diverse taxa, highlighting the universality of microbial contributions to invasion success. Notably, our findings emphasise the importance of diet-derived factors, such as *Eucalyptus* volatiles, in maintaining gut microbiome stability and preventing dysbiosis—a factor critical for the long-term health and reproductive success of *Gonipterus*.

As invasive pests continue to threaten global forestry and agriculture, integrating microbiome research into pest management strategies presents a promising frontier. Targeting key microbial associates or disrupting beneficial host-microbiome interactions could serve as innovative tools for managing *Gonipterus* and other invasive pest populations, mitigating their impact on forestry and agricultural landscapes worldwide. These strategies, combined with ecological and chemical control measures, offer a holistic approach to addressing the challenges posed by invasive pests in an increasingly interconnected world.

## Supplementary Information

Below is the link to the electronic supplementary material.

ESM 1 (XLSX 73.1 KB)

ESM 2 (DOCX 24.3 KB)

## Data Availability

High-throughput sequence data generated in this study is available through NCBI Sequence Read Archive (https://submit.ncbi.nlm.nih.gov/subs/sra/) under the accession number PRJNA1150032.
