# Supplementary material for: Inside the Belly of the Beast: Exploring the Gut Bacterial Diversity of Gonipterus sp. n. 2
Source: Microb Ecol. 2025 Apr 12;88:27. doi: 10.1007/s00248-025-02524-1 (PMC11993490; doi:10.1007/s00248-025-02524-1)
Supplement: Supplementary file 1 [file 248_2025_2524_MOESM2_ESM.docx]

**Supporting Material 1**

**Inside the Belly of the Beast: Exploring the Gut Bacterial Diversity of Gonipterus sp. n. 2**

Rosa S Knoppersen^1^, Tanay Bose^2*^, Teresa A Coutinho^2,3^, Almuth Hammerbacher^1^

^1^Department of Zoology and Entomology, Forestry and Agricultural Biotechnology Institute (FABI), University of Pretoria, Pretoria 0002, South Africa

^2^Department of Biochemistry, Genetics and Microbiology, Forestry and Agricultural Biotechnology Institute (FABI), University of Pretoria, Pretoria 0002, South Africa

^3^Centre for Microbial Ecology and Genomics, University of Pretoria 0002, South Africa

*Corresponding authors – almuth.hammerbacher@fabi.up.ac.za, tanay.bose@fabi.up.ac.za

**Extraction of metabolites from diet samples**

Approximately 40 mg of frozen semi-artificial diet and pulverised *Eucalyptus dunnii* were measured for polar and non-polar GC-MS analysis. To study the non-polar components, 1 ml of hexane (Sigma-Aldrich, USA) was added to each of the eight samples of the artificial diet and *E. dunnii*. Samples were agitated continuously for an hour and centrifuged at 10,000 rpm for 10 min. One ml of the supernatant was then transferred to glass vials. Non-polar extracts were analysed using an Agilent 7890 gas chromatograph equipped with the same previously used GCMS using a 30 m, 0.25 mm, 0.25 µm HP-5ms GC column. Samples were injected in splitless mode. The GC oven gradient was set to 5°C min^-1^ from 40 °C to 325 °C.

The polar components of the artificial diet were initially extracted with 1 ml of methanol (Sigma-Aldrich, USA) followed by an hour of continuous agitation. Methanolic extracts were transferred to a new tube and dried under ambient conditions for approximately two hours. The dried material was resuspended in 100 µl of pyridine (Sigma-Aldrich, USA) containing 20mg.ml^-1^ of methoxamine hydrochloride (Sigma-Aldrich, USA) and 0.05% 2-phenyl-ethanol (Sigma-Aldrich, USA). Samples were incubated at 30°C for 90 min. After incubation, the samples were centrifuged at 12,000 rpm for 20 min. A 30 µl aliquot of the supernatant was transferred into glass inserts containing 30 µl of *N*-Methyl-N-(trimethylsilyl) trifluoroacetamide (MSTFA) (Sigma-Aldrich, USA) within glass vials These solutions were incubated at 37 °C for 30 min and stored at -20°C. Derivatised extracts of the artificial diet and *E. dunnii* were analysed using an Agilent 7890 GCMS using the HP-5ms column. Samples were injected in a 10:1 split ratio. The GC oven was set to 5°C min^-1^ from 70 °C to 325 °C.

**Results:**

**Chemical differences between *E. dunnii* and semi-artificial diet**

The chemicals between the artificial diet and *E. dunnii* from artificial and natural conditions were compared using Gas chromatography coupled with Mass Spectrometry (GCMS). A Principal component analysis (PCA) of *E. dunnii* and the artificial diet illustrated that the biochemical profiles differed substantially from each other. To explore these differences, a table was constructed to determine how the concentrations of major *Eucalyptus* compounds differed (Supplementary Table 4). From the table, the semi-artificial diet exhibited much higher levels of carbohydrates, such as sucrose, d-glucose and d-Talose (Fig. 2; Supplementary Table 4). While high in carbohydrates, the semi-artificial diet had much lower concentrations of monoterpenes (i.e. eucalyptol, α-pinene, d-limonene) (Fig. 2; Supplementary Table 4). In comparison, *E.dunnii* had elevated levels of carbohydrates like that of d-fructose, terpenoids and organic acids, such as Shikimic and Gallic acid (Fig. 2; Supplementary Table 4).

**Supplementary Table 4** List of *Eucalyptus* metabolites across *Eucalyptus dunnii* and the Artificial diet from the ‘diet’ group.

| Compound | **Sample1** | **Sample2** | **Sample3** | **Sample4** | **Sample5** | **Sample6** | **Sample7** | **Sample8** |
| --- | --- | --- | --- | --- | --- | --- | --- | --- |
|  | *E. dunnii* | *E. dunnii* | *E. dunnii* | *E. dunnii* | Artificial diet | Artificial diet | Artificial diet | Artificial diet |
| eucalyptol | 1837958 | 1677740 | 1643092 | 1712465 | 80559.79 | 74733.6 | 56144.49 | 68423.21 |
| *d*-limonene | 138664.4 | 80513.06 | 120914.3 | 133102.2 | 0 | 0 | 0 | 0 |
| *α*-pinene | 1384910 | 1297652 | 1294361 | 1378052 | 35166.91 | 23219.26 | 19328.73 | 12482.55 |
| aromandendrene | 139753 | 134186 | 133396 | 127384 | 23749 | 12819 | 9017 | 4994 |
| globulol | 66596.41 | 65592.65 | 66086.44 | 5992 | 12136.46 | 6768.52 | 6490.35 | 0 |
| *γ*-terpinene | 0 | 0 | 0 | 0 | 4086.36 | 2392.52 | 2761.05 | 0 |
| shikimic Acid | 2135718 | 2475605 | 2353420 | 3452005 | 553371 | 596044 | 874262 | 0 |
| *d*-fructose | 218644 | 250697.2 | 234416 | 293273.6 | 843596 | 985679 | 1399198 | 576344 |
| *l*-(-)-sorbose | 24069 | 0 | 0 | 0 | 485642 | 0 | 807132 | 586121 |
| *d*-(-)-fructose | 122767 | 151195.9 | 145411.8 | 0 | 485450 | 508112 | 0 | 0 |
| *d*-galactose | 0 | 0 | 0 | 0 | 36662 | 32146 | 37069 | 0 |
| *d*-glucose | 252849 | 286901.6 | 273125.5 | 320934.3 | 435170 | 499471 | 716236 | 489345 |
| *d*-(+)-Talose | 0 | 0 | 0 | 0 | 3743005 | 4219062 | 8487624 | 5589859 |
| gallic acid | 2009564 | 2133337 | 1897594 | 2579062 | 846370 | 964106 | 1734877 | 374669 |
| myo-inositol | 613652 | 655420 | 265892.1 | 681302 | 1188794 | 1152892 | 1785684 | 1511883 |
| sucrose | 2550999 | 2898043 | 2518694 | 3059228 | 8318551 | 10177806 | 37380081 | 19519868 |
